# Under pressure: lessons from and beyond entrapment neuropathies

**DOI:** 10.1097/j.pain.0000000000004012

**Published:** 2026-11-02

**Authors:** Annina B. Schmid, Pao-Sheng Chang, Eleonora Maria Camerone, Eva Sierra-Silvestre

**Affiliations:** Nuffield Department of Clinical Neurosciences, University of Oxford, Oxford, United Kingdom

## Abstract

Entrapment neuropathies are the most prevalent focal neuropathic pain conditions globally and impose a substantial individual and socioeconomic burden. Traditionally conceptualised as purely mechanical compression syndromes, this view is increasingly challenged by converging evidence from human tissue analyses, advanced neuroimaging, and deep clinical phenotyping. Entrapment neuropathies are now recognised as biologically dynamic disorders shaped to different extents by microvascular compromise, demyelination, axonal degeneration, immune dysregulation, neuroinflammation, central nervous system adaptations, and psychosocial influences. Varying combinations and dominance of biopsychosocial processes give rise to marked clinical heterogeneity even within the same diagnostic label. This mechanistic diversity helps explain common diagnostic discordances, including extraterritorial symptom spread, normal electrodiagnostic findings despite clinical symptoms indicative of entrapment neuropathies, and imaging–clinical presentation mismatch. It also challenges one-size-fits-all treatment approaches, which have largely failed in entrapment neuropathies.

Uniquely, entrapment neuropathies provide a natural human model of focal nerve injury in the context of neuropathic pain. Surgical decompression allows integration of tissue-level biology with precise clinical phenotypes and offers time-locked insights into recovery. Lessons derived from this model extend beyond entrapment neuropathies and can inform broader understanding of neuropathic and nerve-related musculoskeletal pain conditions. Here, we synthesise contemporary mechanistic insights, examine how biological heterogeneity translates into distinct clinical phenotypes, and discuss potential implications for mechanism-informed management.

## 1. Introduction

Entrapment neuropathies are caused by compression and/or irritation of peripheral nerves as they travel through confined anatomical spaces. Common conditions include carpal tunnel syndrome, cubital tunnel syndrome, Morton's neuroma as well as cervical and lumbar radicular pain and radiculopathies. The most common entrapment neuropathy is carpal tunnel syndrome, with a global estimated prevalence of 14% (95% CI: 0.6%-28%).^37^ The lifetime prevalence of spine-related leg pain (“sciatica”) varies widely from 1.2% to 43%,^70^ likely due to distinct populations studied and different case definitions used (eg, true radiculopathy with neurological deficits vs radiating leg pain).^53^ Entrapment neuropathies place a significant burden on patients due to pain, paraesthesia as well as motor deficits significantly affecting function in the more advanced stages. As they predominantly affect a working population, socioeconomic consequences to both patients and society are considerable. For instance, productivity cost of sickness absence per patient in Finland in the year preceding surgery are €9452 for carpal tunnel syndrome and €8553 for radicular pain, which is higher than those for knee replacement surgeries (€7678).^51^ Estimated annual costs to the healthcare system exceed US$ 2 billion for carpal tunnel syndrome in the United States^130^ and £3.2 billion for low back pain or sciatica in the United Kingdom.^158^

Although most entrapment neuropathies are idiopathic, many shared risk factors have been described, including obesity,^105,126,149^ occupational or physical demands,^74,105^ and comorbidities (eg, diabetes, hypothyroidism).^100,105,107^ Genetic factors also play an important role,^78,152^ with the main genetic influence exerted through genes involved in the size, integrity, and organisation of tissues surrounding affected nerves (eg, muscle or bone),^104^ representing the tunnels in which nerves can be compressed or irritated.

The high prevalence of entrapment neuropathies and associated significant socio-economic challenges justify intensive research efforts. In addition, entrapment neuropathies represent a prototypical model system, as treatment can involve surgical decompression. This offers an unparalleled opportunity to study nerve injury and subsequent regeneration from a clearly defined timepoint. Notably, surgery enables the collection of tissues near the affected nerve (eg, tenosynovium), and in some entrapment neuropathies (eg, Morton's neuroma), even unique access to the affected nerve itself. Unlike postmortem tissue donation, this permits rapid sample processing for downstream analyses, and critically, direct integration with a precise clinical phenotype. Furthermore, entrapment neuropathies often allow comparison with an unaffected contralateral side and typically present with fewer systemic comorbidities that might confound mechanistic interpretation. These advantages are rarely possible in systemic polyneuropathies.

As such, entrapment neuropathies represent a natural model of human nerve injury and neuropathic pain. The study of these conditions has generated substantial mechanistic and diagnostic insights, with implications for management considerations. In the following sections, we distil key mechanistic and clinical insights from entrapment neuropathies and consider their translational relevance including to other (neuropathic) disorders.

## 2. Entrapment neuropathies as multidimensional entities

Historically conceptualised as purely mechanical compression syndromes, entrapment neuropathies are now recognised as biologically dynamic disorders involving vascular, structural, immune, and central nervous system adaptations that interact with psychosocial processes.^121^

### 2.1. From microvascular compromise to structural injury

Rather than frank structural changes induced by acute and severe preclinical models (eg, nerve transection, chronic constriction injury) which are traditionally used to study neuropathic pain,^60^ early entrapment neuropathies are commonly attributed to microcirculatory disturbances resulting from elevated tissue pressures acting on the affected nerves.^21,38^ This is consistent with the typically rapid symptom relief on hand shaking and reversible conduction block in people with mild carpal tunnel syndrome.^67^

Nevertheless, prolonged ischaemia may have downstream consequences that are similar to those observed in traditional, severe nerve injury models. Preclinical models mimicking slowly developing peripheral nerve compression confirm that even mild nerve compression is sufficient to induce extensive demyelination,^44,120^ progressive axonal degeneration predominantly of small nerve fibres,^54,120^ as well as disruption of the blood nerve barrier,^83,154^ resulting in intraneural and extraneural immune dysregulation both locally^42,43,120^ as well as distally and proximally along the neuraxis.^19,120^

Analysing Morton's neuroma, we recently confirmed extensive demyelination compared with control nerves (resected during eg, nerve graft, muscle flap surgery).^116^ Similarly, we found evidence for myelin compromise as apparent by nodal elongation in skin innervated by the affected median nerve in patients with carpal tunnel syndrome and changes in nodal sodium and potassium channel architecture in Morton's neuroma^18^ and carpal tunnel syndrome.^118^ Such demyelination explains the persistent focal conduction slowing or block in patients with entrapment neuropathies,^118^ which improves after surgical decompression.^12,40^ Ion channel changes may also lead to ectopicity with paroxysmal pain as well as tingling being classical features of different entrapment neuropathies.^116^

Following on from demyelination, axon degeneration is a feature of more advanced stages of entrapment neuropathies. As large fibres were historically believed to be predominantly affected,^82^ routine clinical testing traditionally relies on large fibre (eg, A-beta, A-alpha, A-gamma) testing such as electrodiagnostic tests as well as light touch, reflex, and muscle strength assessment.^86^ There is, however, mounting evidence that small fibres (A-delta, C, and autonomic fibres) are also affected in entrapment neuropathies; clinically, a dysfunction of small fibres is apparent by impairments in thermal detection,^128,136,137^ laser evoked potentials,^8^ and changes to sympathetic function.^3^ On sensory bedside neurological examination, hypoaesthesia to pin prick (small fibres) is equally or even more prevalent than light touch abnormalities (large fibres)^48,72^ (Fig. 1). There is also evidence for structural degeneration of small nerve fibres both from preclinical models of mild nerve compression^54,120^ as well as findings of progressive reduction of intraepidermal nerve fibre density (small fibres) in patients with untreated carpal tunnel syndrome.^118^ Small fibres may be affected early in the course of entrapment neuropathies when large fibres are still conducting normally.^118,133^

**Figure 1.**
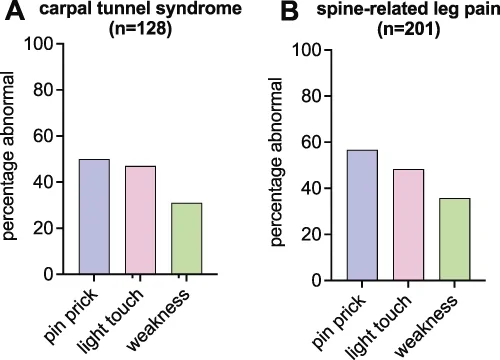
Percentage of abnormal findings during bedside neurological screening in (A) patients with carpal tunnel syndrome and (B) spine-related leg pain (unpublished data from Oxford carpal tunnel syndrome^12,118^ and FORECAST cohorts^122^).

Similar small fibre dominant presentations are also reported in ∼54% of patients with suspected systemic sensory neuropathies.^22^ While predominant compromise of small fibres has been reported in early diabetic neuropathy,^25,146^ parallel changes in both small and large fibres have also been described.^160^

### 2.2. Insights into nerve regeneration

The time-locked initiation of recovery after surgical decompression has been used to understand the regenerative capacity after peripheral nerve injuries. While somatosensory and electrodiagnostic function gradually improve following carpal tunnel and lumbar decompression surgery,^136,145^ most measures remain below normal values even at extended postsurgical timepoints.^12,40,102,136,145^ Similarly, structural regeneration of cutaneous sensory afferents is apparent but fails to reach normal levels 6 months after carpal tunnel surgery.^12^ Of note, the extent of cutaneous small fibre regeneration positively correlated with patients' symptom improvement. Skin analyses in pre and postoperative carpal tunnel syndrome discovered a gene (*ADCYAP1* encoding PACAP) associated with small fibre reinnervation, with its potential as a therapeutic target for human sensory nerve regeneration proven in a humanised in vitro model system.^12^

### 2.3. Non-neuronal contributions

In addition to the abovementioned neuronal changes, intra- and extraneural fibrotic changes are another hallmark of different entrapment neuropathies.^2,20,58,68,116^ Extraneural fibrotic changes are believed to contribute to median nerve tethering^29^ which could explain the reduced gliding of human compressed nerves that is apparent during limb movements in patients with carpal tunnel syndrome and cervical radiculopathy^28,142^ but does not seem to be a defining feature in spine-related leg pain.^111^ Fibrotic signatures were also apparent in Morton's neuroma compared with control nerves.^116^

Recent work increasingly implicates immune cells in entrapment neuropathies. Systemic analyses of blood reveal immune dysregulation in patients with spine-related leg pain^62^ and carpal tunnel syndrome both in the active as well as resolution phases of the disease,^91,114^ although results vary by biomarker. Neural tissue analyses confirm immune contributions in entrapment neuropathies. Bulk transcriptomic analyses in both DRGs compressed by spinal tumours^99^ and Morton's neuroma^116^ suggest the presence of immune dysregulation, that is, chronic inflammatory signatures, in compressed vs control nerves and its association with neuropathic pain. Intriguingly, we identified a specific subtype of glucocorticoid-induced macrophages (M_GC_: MARCO+/CD163+/STAB1+), which was associated with paroxysmal pain in Morton's neuroma.^116^ *MARCO* is also expressed in an over-represented macrophage subcluster in patients with systemic neuropathies,^52^ suggesting translatability of findings from entrapment to other neuropathies. An immune contribution to carpal tunnel syndrome is further apparent from transcriptomic analyses of skin biopsies before vs after surgery^12^ and findings of increased T-cell densities in subsynovial connective tissues surrounding the affected median nerve.^115^ These findings challenge the traditional classification of entrapment neuropathies as noninflammatory disorders and instead position neuro-immune interactions as central contributors to entrapment neuropathies.

While the presence of inflammation may not be a surprise in patients with acute nerve injuries, immune dysregulation notably remains a feature in chronic stages of the disease.^116^ This suggests that inflammation in chronic entrapment neuropathies could be linked to repetitive ischaemia–reperfusion injury-like mechanisms^101^ along with ongoing axonal or myelin degeneration,^12^ or the failure to resolve inflammation once initiated.^32^ The latter is supported by the absence of transcriptomic proresolution markers, such as *Arginase1* and *FoxP3*, in chronic Morton's neuroma.^116^

Noninvasively, microstructural changes to entrapped nerves have been confirmed by advanced neuroimaging methods. Magnetic resonance (MRI) T2 signal intensities are elevated in people with carpal tunnel syndrome^119^ and cubital tunnel syndrome^14^ compared with healthy controls. Furthermore, diffusion-weighted imaging suggests reductions predominantly in fractional anisotropy (a measure of the degree to which water diffusion is directionally dependent) in carpal tunnel syndrome,^112^ cubital tunnel syndrome,^41^ and radicular pain,^106^ which may reflect changes to myelination, axon integrity, or even inflammatory infiltrates.^108,129^ Simultaneous PET and MRI have further demonstrated neuroinflammation in the affected neuroforamina in people with lumbar and cervical radicular pain.^5,81^ In patients with lumbar but not cervical radicular pain, such neuroinflammation is also present along the neuraxis including in the spinal cord^5^ and somatosensory cortex.^7^

### 2.4. Central mechanisms

As mentioned above, neurobiological changes in entrapment neuropathies do not remain localised to the injury site but have been identified within the spinal cord and brain including structural and functional reorganisation.^84,85,95^ The presence of clinically detectable central mechanisms in patients with entrapment neuropathies remains somewhat controversial. Dynamic mechanical allodynia, temporal summation, and secondary thermal hyperalgesia are absent at group level in patients with carpal tunnel syndrome.^128^ However, conditioned pain modulation and widespread pressure hyperalgesia within the forearm and neck have been reported compared with controls.^128^ Data from patients with spine-related leg and arm pain also suggest the presence of widespread hyperalgesia in some patients.^134,135^ Furthermore, symptom spread is common in entrapment neuropathies (see below), more prevalent in patients with moderate/severe neuropathic pain,^90^ and has been attributed to central mechanisms.^123,156,157^

### 2.5. Psychosocial considerations

While well-studied in chronic musculoskeletal conditions, psychosocial factors remain relatively understudied in entrapment neuropathies. Most of our knowledge derives from studies of lumbar radicular pain,^50,71,131^ with a smaller body of literature in cervical radicular pain^88^ and carpal tunnel syndrome.^87,132^

In spine-related leg pain, the influence of psychosocial factors on prognosis is largely understudied. However, high nonverbal pain behaviour, low scores for direct search for social support, a tendency to ignore pain, and a poor ability to imagine coping with the pain were predictive of poor outcome.^9^ In the largest primary care prognostic study of spine-related leg pain (n = 609),^71^ poorer outcome was independently predicted by longer symptom duration, higher symptom “identity” (more symptoms attributed to the condition), and the belief that the problem would persist for longer, while MRI findings of nerve root compression was not predictive. Additional smaller prognostic studies (trial of epidural glucocorticoid injections for chronic unilateral lumbar radiculopathy, N = 116^59^; hospitalised patients with acute severe sciatica, N = 151^33^) highlighted the potential prognostic value of higher education and full-time employment (independently predicting reduced leg pain at 52 weeks) and lower fear avoidance beliefs (predicting better function and less back pain).^59^ There are also some suggestions that unemployment is associated with worse leg pain at 12 months.^33^ Altogether, these findings highlight the need to look beyond structural pathology in lumbar radiculopathy and consider psychosocial factors, including illness perceptions, expectations about pain trajectory, and social determinants of health (eg, education, employment).

In cervical radiculopathy, a systematic review^88^ suggests that depressive symptoms might be associated with worse pain and disability; however, findings were mixed. Anxiety and distress showed similar associations, but few studies were available and inconsistency was high. The authors concluded that mental health symptoms may influence outcomes in cervical radiculopathy, but higher-quality research is needed.

In carpal tunnel syndrome, a systematic review^87^ reported a positive association between psychosocial factors (ie, high psychological distress, job strain, high work demand, low job control, and low social support) and the presence of carpal tunnel syndrome, while greater decision latitude and social support seemed protective. However, inconsistencies were observed across the 6 analysed studies. In a more recent large cross-sectional study of 674 patients with CTS scheduled for surgery, Sun et al.^132^ found that psychosocial factors (eg, psychological distress, pain catastrophizing, and illness perceptions) explained an additional 20% to 25% of variance in CTS severity beyond clinical variables (eg, demographic characteristics, duration of symptoms, and comorbidities), with illness identity showing the strongest association. These findings suggest that how patients interpret and emotionally respond to CTS substantially influences their reported symptom burden.

While the literature remains sparse and currently available studies have only performed limited psychosocial profiling, current evidence underscores the importance of incorporating psychosocial factors to better understand entrapment neuropathies. Potential cause and effect relationships in entrapment neuropathies remain understudied.

## 3. Mechanistic heterogeneity is reflected clinically

From the above, it is readily apparent that the neurobiology underlying entrapment neuropathies is multifactorial, involving both functional and structural changes that can be both transient and sustained. This mechanistic heterogeneity is reflected in patient presentations that differ considerably even within the same diagnostic label. In the following section, we outline key manifestations of this clinical heterogeneity and consider how they reflect underlying mechanistic diversity, both informing and at times challenging diagnostic interpretation.

### 3.1. Symptom distributions may not follow anatomical borders

While classical textbooks describe that neuropathic symptoms should be confined to innervation territories of the affected nerves (eg, dermatomal or peripheral innervation territory), the reality is that approximately 2-thirds of patients with entrapment neuropathies present with extraterritorial symptoms.^16,94^ For instance, up to 2-thirds of patients with carpal tunnel syndrome report tingling in a glove distribution^16^ and 40 to 50% report proximal spread of symptoms into the arm, shoulder, or neck.^156,157^ Similarly, symptoms in patients with lumbar and cervical radicular pain often extend well beyond defined dermatomes.^49,89,94^ While such spread of symptoms may be attributed to variations in innervation territories^77^ and contribution of deeper structures including myotomes or sclerotomes, recent work highlighted a potential involvement of immune dysregulation in symptom spread (see https://www.youtube.com/watch?v=BZYtAR4zUpg). Indeed, patients with carpal tunnel syndrome with proximal symptom spread have serologically elevated C-reactive protein.^161^ Furthermore, PET/MRI scans find indications for neuroinflammation in the somatosensory cortex of patients with radicular leg pain, which is associated with the presence of widespread symptoms documented by the fibromyalgia survey score.^7^

Over-reliance on classically described dermatomal/peripheral innervation patterns should therefore be avoided in the diagnostic work-up; this has recently been recognised as part of the revised neuropathic pain grading system for spine-related leg pain.^125^

### 3.2. Electrodiagnostic and neurological bedside tests can be normal

Electrodiagnostic tests including nerve conduction or needle EMG are commonly used to confirm the presence of a neuropathy and identify its location.^138^ Although these modalities are particularly informative in distal large-fiber neuropathies, findings may be entirely normal in a subset of patients despite clinical features indicating the presence of a peripheral neuropathy. Indeed, approximately 25% of patients with a clear clinical picture of carpal tunnel syndrome have negative electrodiagnostic test outcomes.^153^ Nevertheless, some of these patients still have a confirmed nerve injury as apparent by reduced intraepidermal nerve fibre density in the median nerve territory of the hand,^118^ suggestive of predominant (or early) degeneration of small nerve fibres in a subset of patients.

This work has highlighted the importance of comprehensive neurological testing, including both large and small fibre tests. Of note however, a substantial proportion of patients with suspected entrapment neuropathies have normal neurological examination. For instance, in patients with carpal tunnel syndrome or clinically diagnosed “sciatica”, only 31% and 35.8% have muscle weakness and 47% to 50% have sensory hypoaesthesia (Fig. 1). Only 24.6% of patients with “sciatica” have weaker or absent reflexes.^72^ While this may partly reflect limited sensitivities of neurological bedside tests,^140,155^ more accurate quantitative sensory detection thresholds are also within normal limits in a subgroup of patients.^12,34,118^ The preserved neurological conductivity in some patients with clinical symptoms of entrapment neuropathies may thus reflect the presence of dominant pathomechanisms (eg, immune dysregulation, changes in ion channels) leading to gain rather than (measurable) loss of function.

### 3.3. Neurodynamic test outcomes differ

Neurodynamic tests such as the straight leg raise or slump test are commonly used in the diagnostic work-up of entrapment neuropathies. These tests assess the presence of heightened neural mechanosensitivity by examining the pain response during elongation of the nerve bed.^45^ While increased nerve mechanosensitivity to pressure and stretch can be induced by intraneural changes such as axonal transport disruption,^23^ epineural irritation of nociceptors (nervi nervorum) seems sufficient to induce heightened nerve mechanosensitivity in preclinical models.^24^ Such epineural irritation with activation of nervi nervorum may explain why pain in patients with heightened nerve mechanosensitivity is mostly classed as nociceptive rather than neuropathic.^63^

However, evidence is mounting that these tests have limited diagnostic performance to diagnose entrapment neuropathies.^4,92,147^ Indeed, 54% of patients with CTS^10^ and around a quarter of patients with spine-related leg pain^72^ have negative neurodynamic tests. Intriguingly, the literature consistently shows that patients with entrapment neuropathies as well as systemic neuropathies (eg, painful diabetic neuropathy) who have negative neurodynamic tests have in fact a more severe neuropathy (defined by more extensive loss of neural function).^10,15,73^ It has been speculated that more severe neuropathies inducing degeneration of nervi nervorum may explain this counter-intuitive finding.^10^ Of clinical relevance, a negative neurodynamic test does not exclude the presence of nerve involvement.

### 3.4. Not all patients have neuropathic pain

Neuropathic pain is defined as pain caused by a lesion or disease of the somatosensory nervous system.^57^ Clinically, the presence of neuropathic pain is determined with screening questionnaires or the neuropathic pain grading scale,^31^ which has recently been adapted for patients with spine-related limb pain.^125^ Paradoxically, pain in patients with entrapment neuropathies is not always neuropathic. For instance, 20% of patients with carpal tunnel syndrome have unlikely neuropathic pain.^90^ In patients with a clinical diagnosis of “sciatica”, 48% are unlikely to have neuropathic pain (according to s-LANSS).^72^ This reflects findings in our FORECAST study where 35-43% are classed as unlikely neuropathic pain by the DN4 and painDETECT tools, respectively, while 55% are classed as at least probable neuropathic pain using the grading system (data in preparation).

Patients with neuropathic pain have higher symptom severity and functional compromise, lower emotional wellbeing, and more impaired sleep than those with unlikely neuropathic pain.^47,90^ While somatosensory dysfunction is more pronounced with the presence and increasing severity of neuropathic pain, no differences were apparent in the extent of the neuropathy as defined by electrodiagnostic tests severity and cutaneous innervation.^90^ Patients with persistent neuropathic spine-related leg pain have worse outcomes than those without neuropathic pain^46^ with female sex and higher leg pain intensity showing potential associations with persistent neuropathic pain.^46^

These observations from entrapment neuropathies are similar to those in patients with diabetic neuropathies. For instance, only 15% to 20% of patients with diabetic neuropathy have neuropathic pain.^17^ Those with neuropathic pain also report greater disability, poorer quality of life, and emotional wellbeing, with higher levels of anxiety and depression associated with lower sleep quality.^96,97,109,141^ Similar to carpal tunnel syndrome, patients with painful diabetic neuropathy generally show greater loss of somatosensory nerve function^141^ but electrodiagnostic testing and cutaneous innervations do not show clear differences between patients with painful vs nonpainful diabetic neuropathy.^109,141,144^

As such, the absence of neuropathic pain despite nerve compromise might be due to predominant loss (eg, more severe neuropathy) rather than gain of function mechanisms, or changes predominantly affecting the epineurium rather than the intraneural structures, resulting in nociceptive rather than neuropathic pain.

### 3.5. Conventional imaging–clinical discordance

Imaging modalities are often used to localise entrapment neuropathies. While ultrasound has good diagnostic accuracy for distal entrapment neuropathies including cubital^4^ and carpal tunnel syndrome,^79^ its utility to visualise deeper neural structures in proximal entrapment neuropathies is limited. Instead, MRI is commonly used to diagnose proximal entrapment neuropathies including radiculopathies. However, its diagnostic validity remains unclear^139^ with a substantial proportion of symptomatic patients lacking evidence for nerve root compression on MRI (∼39% with “sciatica,”^72^ 13-15% with cervical radiculopathy^75^). Furthermore, nerve root compromise is also found in asymptomatic segments.^148^

Reasons for this imaging–clinical discordance are manyfold. First, structural changes on MRI may represent normal aging and do not necessarily relate to clinical symptomatology. For instance, neural structures may physiologically adapt to gradual narrowing (eg, such as seen in patients with significant foraminal stenosis but no symptoms) and some people may be more resilient to neural compromise. Such potential protective factors need to be better understood. Furthermore, clinical spine MRIs are routinely performed in a relaxed supine position, but MRIs in more functional positions seem superior to detect neural compromise.^56,151^ Furthermore, traditionally used MRI sequences have limited spatial resolution and thus focus on extraneural macrostructural changes obstructing neural tissues (eg, discs, foramen). Early and predominantly ischaemic and reversible nerve problems may not be characterised by microstructurally detectable changes.

Microstructural intraneural changes may well provide more detailed insights into neural compromise. Diffusion tensor imaging of peripheral nerves has gained increasing interest over the past decade. Fractional anisotropy is lower in symptomatic vs asymptomatic nerve roots in patients with radicular pain or radiculopathy.^106^ Lower fractional anisotropy is also a feature of cubital^41^ and carpal tunnel syndrome^112^ as well as more difficult to diagnose entrapment neuropathies such as pudendal neuropathy.^27^ Notably, diffusion metrics moderately to strongly correlate with patients' symptoms or disability across a range of peripheral neuropathies^55,106,119^ or with functional neuronal measures such as electrodiagnostics,^65^ distinguishing them from conventional macrostructural imaging markers. Together, these data suggest that conventional macrostructural imaging captures only a subset of nerve pathology while advanced neuroimaging may be promising to detect symptom-relevant intraneural microstructural changes.

### 3.6. Beyond entrapment: minor nerve injury in traditionally musculoskeletal conditions

Given the above, it is evident that detecting minor nerve injuries such as entrapment neuropathies particularly in the absence of frank neuronal degeneration (such as in traumatic nerve injury) can be challenging. It has been hypothesised that excessive nerve compression or tension leading to minor nerve injuries may be a feature of other musculoskeletal conditions, that are traditionally viewed as non-neuropathic. The most recent example is whiplash-associated disorder grade II, which is defined as complaints of neck pain with musculoskeletal signs such as decreased range of motion and point tenderness in the absence of neurological signs.^103^ An increasing number of studies consistently identified neuronal compromise in a subset of patients diagnosed with whiplash-associated disorder grade II through somatosensory profiling, advanced neuroimaging, skin biopsies, and serologic markers of axonal injury (neurofilament light chain).^30,35,36,110^ These observations raise the possibility that subtle nerve injury and neuroinflammation may contribute to symptom persistence in conditions historically conceptualised as purely musculoskeletal.

## 4. A clinical framework to unpick clinical heterogeneity

The above discussed diagnostic discordance across several assessments is likely a reflection of mechanistic diversity within the same clinical diagnosis. To help clinicians identify treatment relevant subgroups, we have described a mechanism-based clinical framework which operationalises mechanistic heterogeneity into clinically observable phenotypes with the ultimate goal to guide targeted management (Fig. 2).^124^ This framework integrates the terminology recommended for spine-related leg pain by the Neuropathic Pain Special Interest Group^125^ with the pain mechanisms described by the International Association for the Study of Pain^1^ and has shown good intra- and intertester reliability in a clinical context.^64^ The framework distinguishes subgroups characterised by neuronal gain of function (ie, neuronal hyperexcitability anywhere along the neuraxis) and loss of function (ie, loss of action potential conductivity anywhere along the neuraxis).

**Figure 2.**
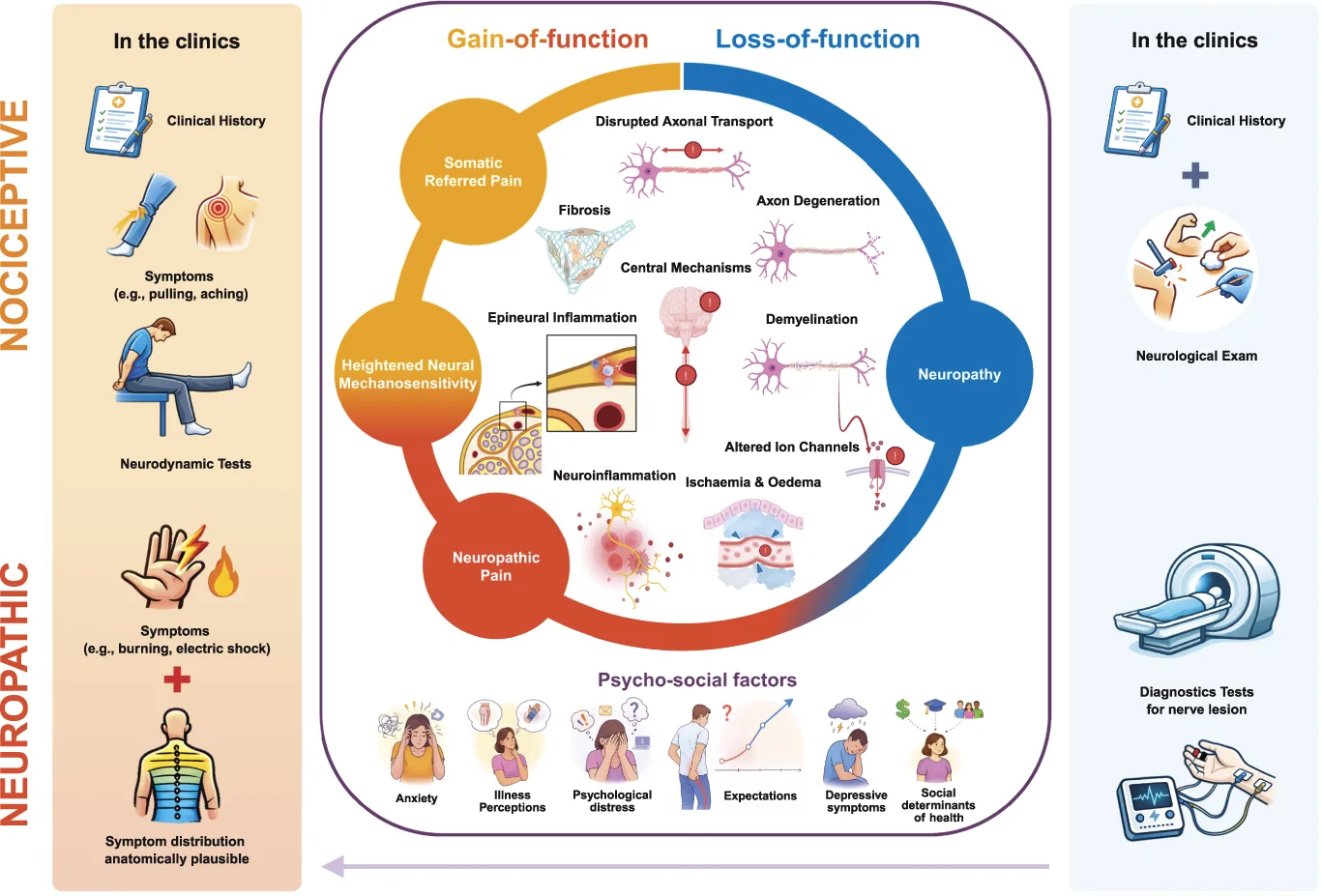
Mechanism-based clinical framework which operationalises mechanistic heterogeneity into clinically observable phenotypes. This figure shows the continuum between gain of function (left, orange and red) and loss of function (right, blue) mechanisms contributing to different clinical presentations. The neurobiological mechanisms associated with the clinical phenotypes are depicted within the circle. Some mechanisms predominantly drive loss of function (eg, axon degeneration, demyelination), and others gain of function (eg, intraneural and epineural inflammation). Nevertheless, these mechanisms can coexist, are interdependent, and interact, explaining the frequent coexistence of clinical phenotypes. Psychosocial factors interact with biological processes to influence symptom experience across phenotypes. In clinical practice, the neuropathic pain grading system can be used to differentiate somatic referred from neuropathic pain. This system integrates information from clinical history with symptom characteristics and distribution (left). The presence of a neuropathy is determined by a neurological examination and if required, the presence of a diagnostic test showing the nerve lesion (right). The presence of a neuropathy increases the likelihood of neuropathic pain (arrow). Neurodynamic tests are used to assess heightened neural mechanosensitivity.

### 4.1. Loss of function: neuropathy

Neuropathy represents the loss of function phenotype which is characterised by the presence of a neurological deficit (eg, sensory loss, reduced or absent tendon reflexes, or muscle weakness). Bedside assessments to identify the neuropathy subgroup include a thorough neurological examination, but diagnostic tests such as electrodiagnostic testing can also provide information about loss of neuronal function. Common neuropathy phenotypes include median neuropathies (eg, loss of neuronal function caused by carpal tunnel syndrome) as well as radiculopathies (loss of neuronal function caused by nerve root compromise).^125^ While these presentations are often combined with a gain of function (see below), painless entrapment neuropathy does exist similar to painless diabetic neuropathy.^141^ An example is a painless foot drop (eg, in pure ventral root radiculopathy or in late stages of radiculopathy where pain may have resolved) or a suprascapular nerve palsy which often presents with infraspinatus weakness in the absence of pain.

### 4.2. Gain of function: somatic referred vs neuropathic pain

On one side of the gain of function spectrum are patients with somatic referred (nociceptive) pain, which is initiated and maintained by noxious stimulation of nerve endings in somatic structures (eg, cartilage, joints, muscles, tendons, ligaments, fascia, and bones).^125^ Somatic referred pain is usually attributed to convergence of wide dynamic range neurons in the spinal cord resulting in pain perceived in a different somatic area than the actual lesion site.^61^ A typical presentation of somatic referred pain is a person with discogenic low back pain experiencing a diffuse, dull annoying pain in their leg.

This contrasts with the second gain of function presentation, neuropathic pain which is caused by a lesion or disease of the somatosensory nervous system. These 2 pain phenotypes, somatic referred and neuropathic, are clinically differentiated with the neuropathic pain grading system.^31^ Rather than a straight-forward yes/no decision, determining the presence of neuropathic pain represents a nuanced certainty rating (ie, unlikely, possible, probable, definite neuropathic pain). The presence of a neuropathy (ie, loss of sensory function) increases the certainty of the presence of neuropathic pain and as such these 2 groups are interlinked. A typical example of a patient with definite neuropathic pain is a patient with a painful cervical radiculopathy who experiences burning pain and tingling in their arm accompanied by sensory loss in the same anatomical area and an MRI scan confirming a nerve root compromise concordant with the clinical presentation.

### 4.3. Gain of function: heightened neural mechanosensitivity

The third gain of function subgroup includes patients who present with heightened neural mechanosensitivity on pressure or tension of the nervous system (for instance assessed with neurodynamic tests). While heightened neural mechanosensitivity is not part of IASP or NeuPSIG terminology,^1,125^ we include this subgroup due to the consistently reported distinct test outcomes in patients^10,72,73^ and increasing evidence for therapeutic relevance for this subgroup (see below) particularly in entrapment neuropathies.

### 4.4. Mixed phenotypes

It is increasingly recognized that most patients present with a mixed pain phenotype; 77% of patients with spine-related arm pain^63^ and 96.5% of patients with spine-related leg pain are classified into 2 or more phenotypic groups (^143^in preparation). The most common combinations are somatic referred pain with heightened neural mechanosensitivity^63^ and neuropathy (loss-of-function) with neuropathic pain and heightened neural mechanosensitivity (^143^in preparation). While psychosocial contributions have been reported to be more pronounced in neuropathic pain, they can be a feature of all mechanistic subgroups.

Clarifying the individual “pain mix,” and whether a particular mechanism predominates, may be therapeutically consequential. Notably, presentations clustering toward the bottom right of the framework likely carry more weight for management decisions. For instance, a patient with a radiculopathy and progressive neurological deficit may require surgery, and the presence of neuropathic pain is associated with a higher health impact than non-neuropathic pain.^11^

## 5. Management consequences

International guidelines suggest conservative management consisting of physiotherapy and pharmacology as first-line interventions for entrapment neuropathies with surgical options considered if conservative management fails or if progressive/severe neurological deficits exist.^66,98^ However, systematic review evidence for the most prevalent entrapment neuropathies suggest that conservative treatments have only limited benefit compared with minimal interventions^26^ and may be inferior to surgical management.^69,80^ Although this information must be interpreted in the light of largely low quality of evidence and balanced with higher side effects and complication rates of surgery.

Nevertheless, the clinical trial literature is overwhelmingly consistent; a one-size-fits-all approach fails in the management of entrapment neuropathies. Considering their well-recognised clinical and mechanistic heterogeneity, there is increasing advocacy for more targeted, stratified therapeutic approaches. Indeed, we have shown that most studies using neuropathic pain medication for low back and spine-related leg pain fail to adequately determine the presence of neuropathic pain.^150^ We identified a trend toward larger effect sizes (albeit moderate to small) for pain relief and improvements in disability in people with definite/probable neuropathic pain compared with negligible to small effects in those with unlikely or lower certainty of neuropathic pain. However, certainty of evidence was low to very low and future prospective studies with careful pain phenotyping are thus needed to determine whether patients with neuropathic pain are indeed more likely to benefit from neuropathic pain medications.^150^

Another example illustrating the potential promise of phenotyping patients according to the framework concerns neural mobilisation interventions. These have shown effectiveness for patients with spine-related leg and arm pain as well as carpal tunnel syndrome.^13,76,93,159^ Applying the same scrutiny to understanding the patient population studied in these trials, it becomes apparent that the inclusion criteria used to define spine-related limb pain in these studies heavily relied on the presence of heightened neural mechanosensitivity.^76,93^ A total of 83.3% of studies in spine-related leg pain^93^ and 80.8% of studies in spine-related arm pain^76^ included heightened nerve mechanosensitivity as part of their inclusion criteria (Fig. 3). This trial evidence, which is limited by low-quality evidence, is complemented by a cohort study demonstrating a more favourable response to interventions in patients with spine-related leg pain and heightened nerve mechanosensitivity compared with those with somatic referred pain, neuropathic pain, and radiculopathy.^117^

**Figure 3.**
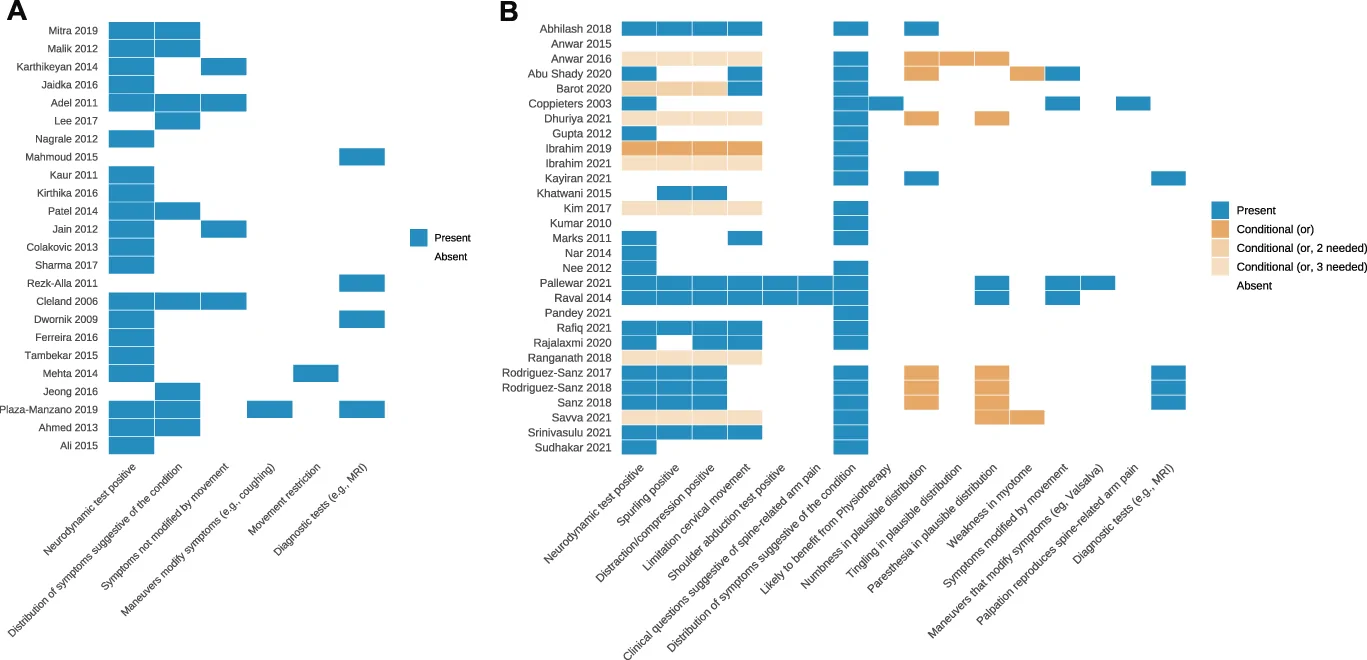
Inclusion criteria used to define (A) spine-related leg pain and (B) spine-related arm pain in trials studying the effectiveness of neurodynamic treatments. Colour coding: blue: Criterion had to be fulfilled for a patient to be included. Orange: Criterion was tested and could be present or absent within a cluster of tests. White: Criterion was not part of the inclusion criteria. Data were extracted from studies included in the systematic reviews by (A) Murape et al.^93^ and (B) Lascurain-Aguirrebena et al.^76^

These signals, albeit preliminary, suggest that the targeted treatments against different clinical phenotypes with distinct underlying pathomechanisms may indeed be more promising than a one-size-fits-all approach. The field now needs large-scale studies (both cohorts and trials) to determine whether personalised management indeed improves efficacy of conservative treatments for patients with distinct patterns of entrapment neuropathies.

Notably, the framework is likely supporting clinicians in better understanding individual patient presentations and reduce their own uncertainties. Diagnostic uncertainty is common in the care of patients with musculoskeletal conditions.^6,127^

The complexity of patients presenting with an entrapment neuropathy requires the clinician to have a deep understanding and knowledge of the multiple factors which may contribute to the patient's presentation. Such knowledge and high levels of clinical reasoning enable the clinician to better support people living with these conditions who often struggle to understand their symptoms.^113^ Currently, there still remains an outdated focus on the mechanisms associated with mechanical compression without taking into account the myriad of factors which cumulate in the individualised presentation of people with entrapment neuropathies.^39^

### 5.1. Conclusion

Entrapment neuropathies demonstrate that focal nerve injury is rarely a purely mechanical phenomenon. Instead, they reveal a multidimensional pathobiology encompassing vascular compromise, demyelination, axonal degeneration, immune dysregulation, central adaptation, and psychosocial modulation. This mechanistic diversity likely explains clinical heterogeneity and potentially differential treatment responses.

Leveraging entrapment neuropathies as a unique natural model system serves as a blueprint for better understanding mechanisms at play in patients with neuropathies and neuropathic pain. The challenge ahead lies in the translation of mechanistic phenotyping into improved care for people with entrapment neuropathies and beyond.

## Conflict of interest statement

The authors have no conflict of interest to declare.
